# An Alpine ant’s behavioural polymorphism: monogyny with and without internest aggression in *Tetramorium alpestre*

**DOI:** 10.1080/03949370.2017.1343868

**Published:** 2017-07-20

**Authors:** Patrick Krapf, Lucia Russo, Wolfgang Arthofer, Markus Möst, Florian M. Steiner, Birgit C. Schlick-Steiner

**Affiliations:** Molecular Ecology Group, Institute of Ecology, University of Innsbruck, Technikerstrasse 25, Innsbruck 6020, Austria

**Keywords:** aggression, behaviour analyses, Formicidae, microsatellites, social structure, *Tetramorium alpestre*

## Abstract

Social structure influences animal societies on various levels (e.g., relatedness, behaviour). In ants, both the number of matings per queen and the number of queens per colony can vary strongly. While workers from both monogynous and polygynous colonies often fight fiercely, in supercolonies (an extreme form of polygyny comprising thousands of queens in spatially separated but interconnected nests), non-nestmates interact peacefully. Studies on social and behavioural polymorphism within ant species can help elucidate their influence on genetic diversity and behaviour and the factors triggering variation in social structure and behaviour. Here, we reveal a behavioural and social polymorphism comprising monogyny with and without internest aggression in *Tetramorium alpestre* sampled in Tyrol, Austria. The social polymorphism is based on genetic and behavioural evidence and contrasts with the supercolonial organisation known from another location in Austria (Carinthia), 150 km away. Microsatellite genotyping using eight polymorphic loci revealed monogyny-monandry and high intranest pairwise relatedness. Interestingly, various experimental one-on-one worker encounters revealed only occasional aggressive behaviour between monogynous colonies, and thus a behavioural polymorphism. Mantel tests revealed a significant negative correlation between spatial distance and relatedness, while worker behaviour was not correlated with relatedness or spatial distance. These results indicate that behaviour might be influenced by other factors – for example, the experience of workers, ecological, chemical, and/or genetic factors not characterised in this study. However, workers distinguished nestmates from non-nestmates also when aggression was lacking. We hypothesise an adaptive value of reduced aggression. We speculate that the non-aggressive and partly aggressive encounters observed represent different options in the social structure of *T. alpestre*, the non-aggressiveness possibly also promoting supercolony development. The social and behavioural polymorphisms observed offer opportunities to identify the factors triggering these changes and thus further explore the behavioural and social polymorphism of this ant species.

## INTRODUCTION

Social structure varies greatly among animal societies. Within each society, the number of reproductive individuals shapes its genetic diversity (Hughes et al. 2008). Ants are among the most abundant and ubiquitous organisms in the world (Alonso 2009). In some ant species, social structure varies, which is termed social polymorphism (Gyllenstrand et al. 2002). Gynes either mate once (monandry) or multiply (polyandry), and males may mate with one or several gynes (Heinze 2008). Colonies are headed by one single queen (monogyny) or by several queens (polygyny; Schmid-Hempel & Crozier 1999), and monogynous and polygynous colonies can live in either one nest (monodomy) or two or more nests simultaneously (polydomy; Crozier & Pamilo 1996).

Supercolonies are an extreme form of polygyny and polydomy. They are extensive cooperative units with many queens and very many workers integrated harmoniously over several square metres to many square kilometres (Crozier & Pamilo 1996; Giraud et al. 2002; Steiner et al. 2009). Supercoloniality is often considered key to the success of invasive ant species (Holway et al. 2002), and supercolonies have been thoroughly studied over the last decades (Tsutsui et al. 2000; Giraud et al. 2002; Pedersen et al. 2006; Leniaud et al. 2011; Huszar et al. 2014; Kennedy et al. 2014). However, the factors triggering their emergence remain largely unknown (Suarez & Suhr 2012).

Beside social polymorphism, behavioural polymorphism can also be observed within ant species describing variation in behaviour. Workers belonging to the same supercolony behave peacefully towards each other, although they may derive from different nests often several hundreds to thousands of kilometres apart (Giraud et al. 2002), while workers of monogynous and polygynous colonies usually fight fiercely when non-nestmates are encountered. However, non-aggressive behaviour between workers from monogynous colonies can also be observed (Steiner et al. 2007 and references therein), and severe aggression occurs between supercolonies (Giraud et al. 2002), indicating that this behaviour is a variable trait.

**Fig. 1.**
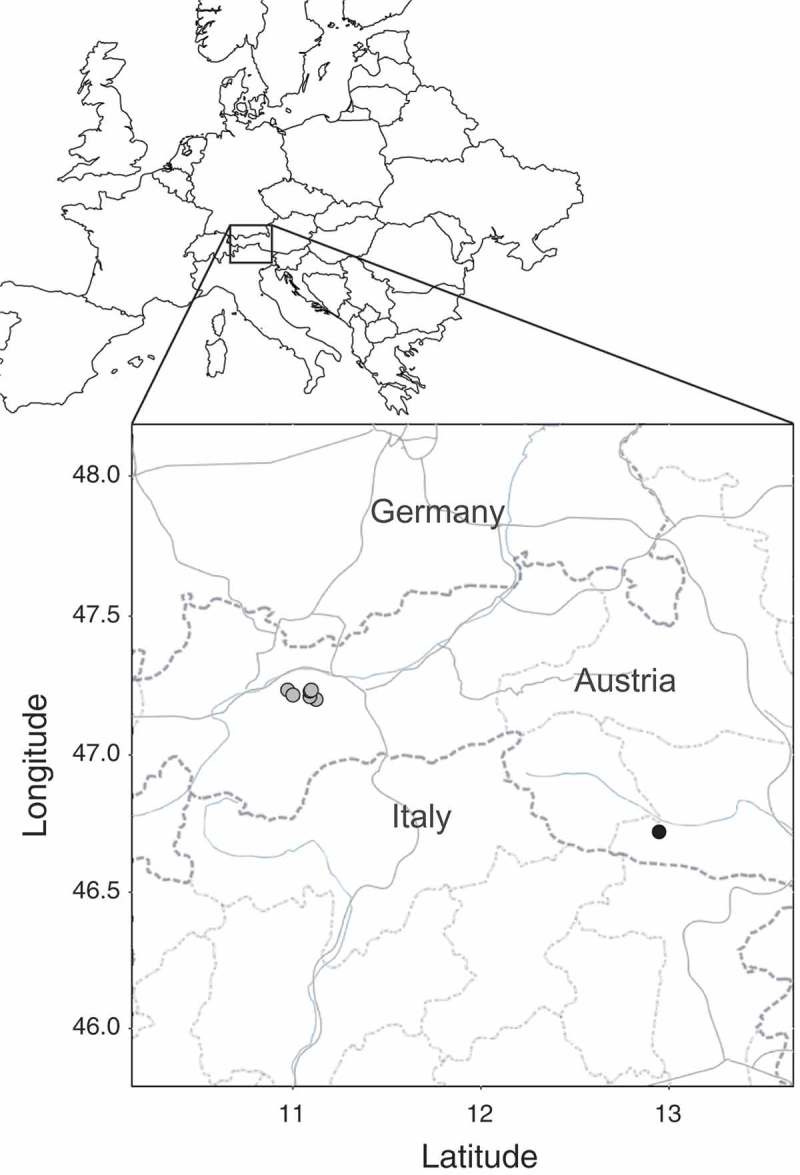
Map of the sampled nests in Tyrol (grey points) and of the location of the known supercolony in Carinthia (black point).

In this pilot study, we investigate the Alpine ant *Tetramorium alpestre* (Steiner et al. 2010), which is known to be supercolonial in Carinthia (Steiner et al. 2003) and thought to form monogynous-monodomous colonies in Tyrol, based on field observations (Fig. 1; F.M. Steiner et al. unpublished data). Clear evidence of monogyny-monodomy in Tyrol and thus confirmation of a social polymorphism within the species would make *T. alpestre* a highly suitable study organism for exploring the ecological and genetic factors triggering transitions from monogyny to polygyny and eventually supercoloniality. We therefore investigated various nests in Tyrol using behavioural analysis and microsatellite genotyping, addressing four questions: (1) Do *T. alpestre* nests differ in their social structure? (2) Do nests differ in their behaviour in terms of aggression between nests? (3) Are worker behaviour, internest averages of pairwise relatedness, and geographic distance correlated? (4) Do workers that are non-aggressive towards workers from other nests discriminate nestmates from non-nestmates?

## MATERIALS AND METHODS

### Fieldwork and worker maintenance

In 2011, workers were collected from 11 nests in Tyrol, Austria (Supplementary Table 1), with distances among nests ranging from 9 m to 9 km (Supplementary Table 2). Workers were killed in 96% ethanol for genetic analyses or collected alive for aggression tests. See Supplementary online material for all details on fieldwork and worker maintenance.

### Microsatellite genotyping and allele analyses

DNA was extracted from 12 workers per nest, that is, a total of 132 workers using the GenElute™ Mammalian Genomic DNA Extraction kit (Sigma-Aldrich, Saint Louis, USA) following the manufacturer’s protocol. The DNA extracts were amplified using primers for nine microsatellites (Steiner et al. 2008). For all details on microsatellite genotyping and allele analyses, see Supplementary online material.

MICROCHECKER v2.2.3 (Van Oosterhout et al. 2004) was used to test for null alleles. Arlequin v3.5 (Excoffier & Lischer 2010) was used to calculate an exact probability test for deviations from linkage equilibrium (LE) and Hardy-Weinberg equilibrium (HWE) using the Markov chain method with default parameters. To decrease the effect of relatedness of individuals, two subsets consisting of either four or one randomly chosen worker(s) per nest were used for these tests (Qian et al. 2012). Arlequin was further used to calculate observed (H_O_) and expected (H_E_) heterozygosities. The distribution of genetic differentiation was analysed among all nests. The fixation index (F_ST_) and the inbreeding coefficient (F_IS_) were calculated as an average over all loci with 10,000 permutations. The F_ST_ matrix for pairwise nest-comparisons was analysed for significant genetic differentiation between nests. GenAlEx v6.502 (Peakall & Smouse 2012) was used to calculate pairwise geographic distances between nests. For all statistics involving multiple pairwise comparisons, a Bonferroni–Holm correction (Holm 1979; Rice 1989) was performed.

A non-detection error (NDE) for each nest was calculated following Boomsma and Ratnieks (1996). The NDE is the probability that two males share the same genotype at all loci by chance, which might cause underestimation of queen-mating frequencies. A non-sampling error (NSE) for each nest was calculated following Foster et al. (1999), using a proportion of offspring of *P* = 0.10. NSE estimates whether sampling size is sufficient to detect all males siring offspring.

COLONY v2.0.6.2 (Jones & Wang 2010) was used to infer sibship using workers’ individual multilocus genotypes. Based on these results, the effective number of matings per queen corrected for sample size (*M*_e,p_) was calculated following Nielsen et al. (2003). The intranest and internest averages of pairwise relatedness (*r*_ww_) were calculated in GenAlEx using the algorithms of Queller and Goodnight (1989). Based on these results, the effective number of queens (*f*) was calculated following Pamilo (1991).

### One-on-one encounters and behaviour analyses

For the behaviour assays, one-on-one encounters were chosen because they represent encounters of single workers foraging distantly from the nest (Roulston et al. 2003), thus mimicking natural conditions. Encounters were performed following Giraud et al. (2002) with modifications (see Supplementary online material for details on one-on-one encounters). Four replicates of each pairwise intra- and internest combination were filmed for 3 min (Roulston et al. 2003). The films were examined in slow motion, and the observer had access to the information of the origin of workers. Such a situation is considered problematic by some researchers, as it might influence the observation and introduce bias (van Wilgenburg & Elgar 2013). However, the observer had no information on the relatedness between workers (Frizzi et al. 2015). The behaviours scored were ignoring (0), being next to each other without contact (1), antennation (2), food exchange or cleaning (3), avoiding (4), mandible threatening (5), biting (6), and fighting (7). Levels 4–7 were categorised as aggressive. The behaviours scored were adapted from those used in similar experimental designs (Giraud et al. 2002; Steiner et al. 2003; Boulay et al. 2007; Charbonneau et al. 2015).

For the aggression-index calculations, only aggressive behaviours were used, while all non-aggressive behaviours were excluded, similar to Boulay et al. (2007). Two indices were calculated: (1) aggression index (AI, modified from d’Ettorre & Heinze 2005); and (2) mean maximum aggression index (MMAI, Vogel et al. 2009). For AI, the frequency of each observed aggressive behaviour per worker was multiplied with its respective scoring level (4–7) and their sum divided by 180, as there were 180 records (one per second). The arithmetic mean of the four replicates was calculated. For MMAI, the arithmetic mean of the highest aggression values observed in each encounter over the four replicates was calculated.

In all statistical analyses, an alpha level of 0.05 was used. Before applying any *t*-test, an *f*-test was performed to check for differences in variances. If variances were significantly different, a modified *t-*test using a Satterthwaite approximation accounting for heteroscedasticity was used. To check for differences in behaviour between intranest and internest encounters, two-sided *t*-tests were calculated in R (R Development Core Team 2016) using the mean behaviour frequencies.

### Mantel and partial mantel tests and recognition test

Mantel tests were calculated to check for correlations between the geographic distance and the behaviour indices (A) AI and, separately, (B) MMAI, internest average of pairwise relatedness and (C) AI and, separately, (D) MMAI, and (E) internest average of pairwise relatedness and geographic distance. Partial Mantel tests (‘Pearson method’) were calculated to check for correlations between geographic distance, internest average of pairwise relatedness, and the behaviour indices AI and, separately, MMAI while controlling for the effect of each of the variables. The Mantel and partial Mantel tests were performed with 9999 permutations using the ‘biotools’ and the ‘ecodist’-package in R, respectively. To determine the power of the Mantel tests for different effect sizes, sensitivity power analyses were performed using the ‘mantelPower’-function in the ‘biotools’-package in R. For these analyses, the effect sizes were set to range from 0.10 to 1.00 incrementing by 0.01, and the respective power was calculated for each effect size (Supplementary Table 3). To assist the interpretation of our results, effect sizes calculated from our data were then compared with the effect sizes that would have yielded significant results (*P* < 0.05) with power set to 0.8, a standard threshold for power analyses. A Spearman rank correlation was performed to check for an association between the intranest average of pairwise relatedness and the mean aggression level of each nest (AI, and, separately, MMAI). A two-sided *t*-test was calculated in R using the total antennation time from intranest and non-aggressive internest encounters to detect differences regarding worker discrimination.

## RESULTS

### Genetic analyses

For both subsets (four workers and one worker from each nest), MICROCHECKER revealed the possible presence of null alleles in locus 55a, which thus was excluded from further analyses. After correction for multiple comparisons, no locus deviated from HWE or departed significantly from LE for both subsets. The eight loci yielded a total of 68 alleles in the 132 workers genotyped. The mean H_O_ value was significantly higher than H_E_ (two-sided *t*-test, *t*_136.73_ = 7.74; *P* < 0.001 for all nests). Among all nests, the global F_ST_ value for internest genetic differentiation was 0.37 (*P *< 0.001); the global F_IS_ value for genetic inbreeding was not significant (– 0.58, *P *= 1.00). After Bonferroni–Holm correction, all pairwise-nest comparisons were significant regarding their F_ST_ values (internest genetic differentiation), which ranged from 0.20 to 0.52 (data not shown).

**Fig. 2.**
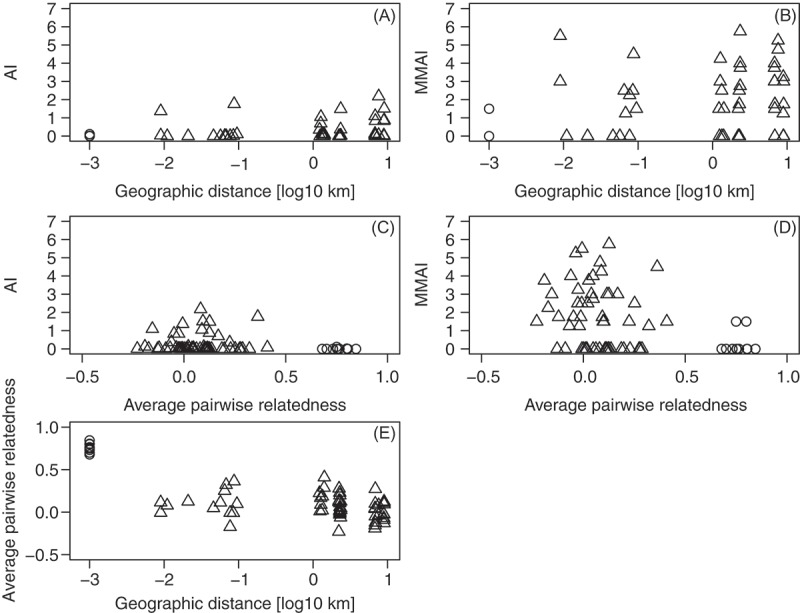
(A) Aggression index values (AI, modified from d’Ettorre & Heinze 2005) and (B) mean maximum aggression index values (MMAI) plotted against log_10_ transformed geographic distance, (C) AI and (D) MMAI plotted against the intranest and internest averages of pairwise relatedness values, and (E) intranest and internest averages of pairwise relatedness plotted against the log_10_ transformed geographic distance. Dots and triangles represent intranest and internest values, respectively. In plots showing the geographic distance, the intranest comparisons are set at – 3 on the x-axis, while internest comparisons are shown at their respective distance. Relatedness values were calculated using the algorithm of Queller and Goodnight (1989).

COLONY assigned 11 monogynous-monandrous colonies to the 11 nests sampled and calculated a number of 11 queens (*Q*_est_) and 11 males (*M*_est_). A *M*_e,p_ value of 1.00 for all nests confirmed that queens mated only once. The NDE ranged from 2.02 × 10^–8^ to 9.97 × 10^–37^ for all nests. The NSE was 1.62 × 10^–3^ for all nests. The averages of pairwise intranest and internest relatedness values ranged from 0.62 to 0.82 and from – 0.39 to 0.31, respectively (Fig. 2E; Supplementary Table 4). Negative relatedness values can be observed if, for example, compared individuals differ in their allele frequencies. Based on the calculation of the effective number of queens (*f*, Supplementary Table 1) and the average pairwise intranest relatedness values, each nest was inferred to have one single queen.

### Aggression tests and behaviour statistics

**Fig. 3.**
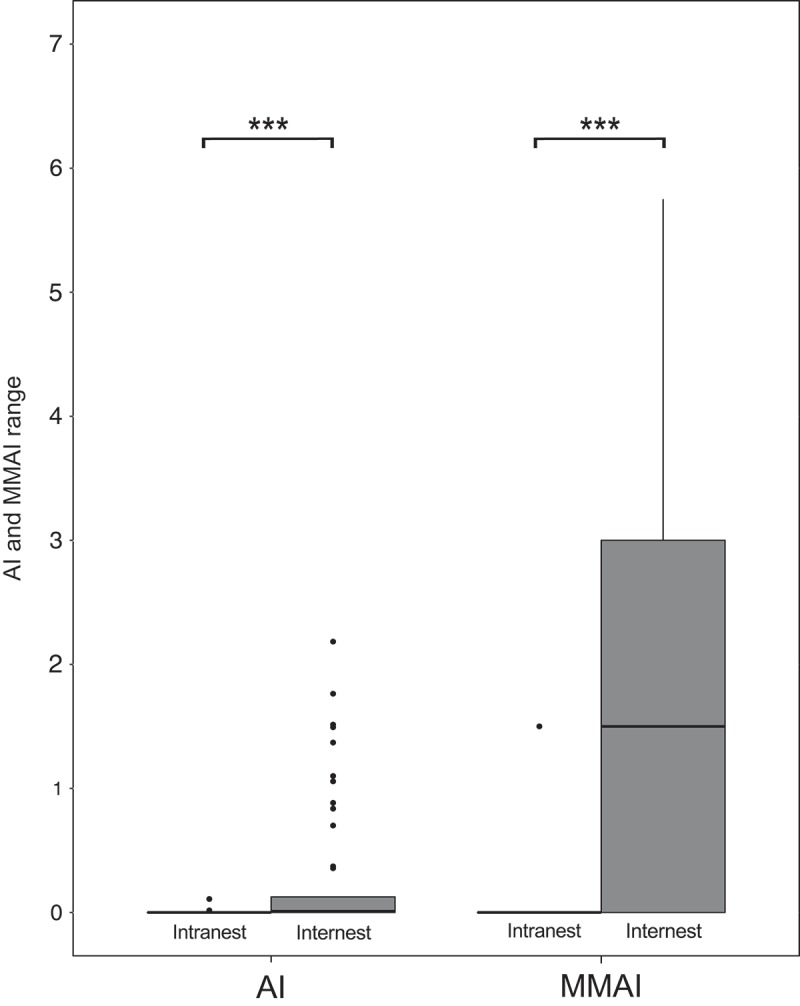
Aggression index (AI) values and mean maximum aggression index (MMAI) values from Tyrol on different organisational scales. Intranest and internest represent the aggression indices observed within and between nests, respectively. Whiskers represent the lowest and highest data still within 1.5 interquartile range of the lower and upper quartile, respectively, and dots represent outliers beyond the 1.5 interquartile range. Asterisks represent significant differences between intranest and internest behaviour levels (two-sided *t*-tests, *** = *P* < 0.001).

**Fig. 4.**
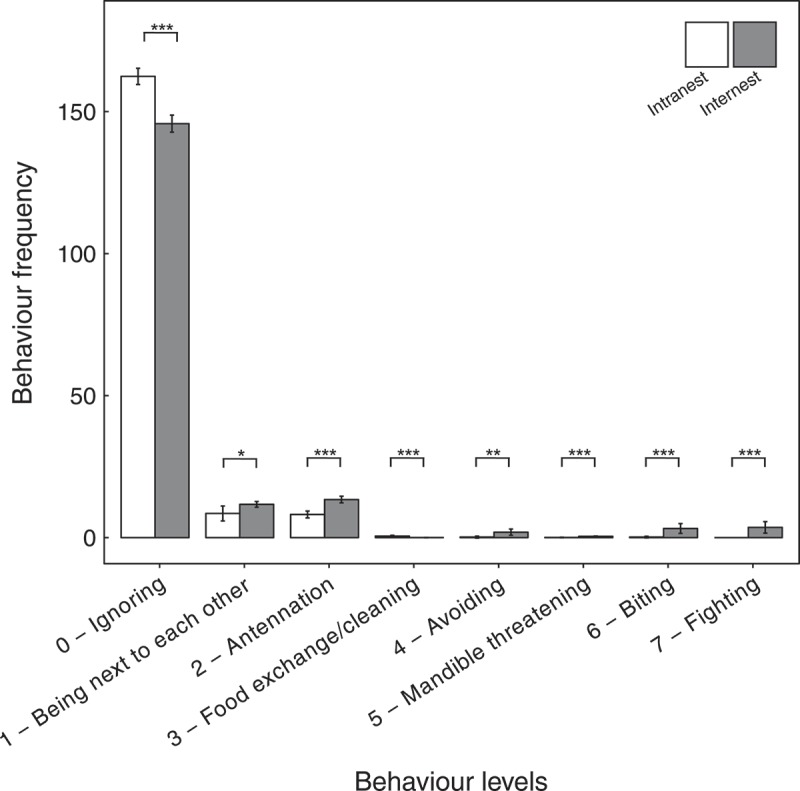
Mean frequency ± 1.96 × standard error of the mean (SE) of all seven behaviours observed. White and grey bars represent intranest and internest encounters, respectively. Asterisks represent significant differences between behaviour levels (two-sided *t*-tests, * = *P* < 0.05, ** = *P *< 0.01, *** =* P* < 0.001).

In the 44 intranest encounters, AI and MMAI ranged from 0.00 to 0.11 and from 0.00 to 1.50, respectively. In the 220 internest encounters, AI and MMAI ranged from 0.00 to 2.18 and from 0.00 to 5.75, respectively (Fig. 2A-B; Supplementary Tables 5–6). Both the AI and MMAI values significantly increased from intranest to internest encounters (two-sided *t*-tests, AI, *t*_55.90_ = – 3.88, *P* < 0.001; MMAI, *t*_46.38_ = – 5.02, *P* < 0.001; Fig. 3). In internest encounters, both non-aggressive (level 0–3) and aggressive (level 4–7) behaviours were detected. In seven of 220 internest encounters, workers were fighting throughout the observation. In 62 encounters, workers were fighting briefly and stopped fighting after some time. In the remaining 151 of 220 encounters, no aggression was observed. However, workers of all colonies reacted at least briefly with aggressive behaviour in at least one pairing. The mean frequencies of behaviour levels 1, 2, 4, 5, 6, and 7 (being next to each other, antennation, avoiding, mandible threatening, biting, and fighting) were significantly higher in internest than in intranest encounters (two-sided *t*-test, level 1, *t*_110.10_ = – 2.02, *P* = 0.046; level 2, *t*_271.02_ = – 5.83, *P* < 0.001; level 4, *t*_507.24_ = – 2.90, *P* = 0.004; level 5, *t*_493.45_ = – 4.19, *P* < 0.001; level 6, *t*_459.88_ = – 3.39, *P* < 0.001; and level 7, *t*_439.00_ = – 3.51, *P* < 0.001), while the mean frequency of behaviour levels 0 and 3 (ignoring and food exchange) were significantly lower in internest than in intranest encounters (two-sided *t*-test, level 0, *t*_304.85_ = 7.60, *P* < 0.001; level 3, *t*_89.34_ = 3.61, *P* < 0.001, Fig. 4; all *t*-tests remained significant after Bonferroni–Holm correction).

### Mantel and partial mantel tests and recognition test

Geographic distance had no influence on AI (Mantel test A, *r* = 0.16, *P* = 0.35, Fig. 2A) and MMAI (Mantel test B, *r* = 0.11, *P* = 0.54, Fig. 2B). Internest averages of pairwise relatedness were not correlated with AI and MMAI (AI, Mantel test C, *r* = – 0.03, *P* = 0.87; MMAI, Mantel test D, *r* = – 0.20, *P* = 0.14, Fig. 2C–D). Geographic distance was significantly negatively correlated with the average of pairwise relatedness (Mantel test E, *r* = – 0.31, *P* < 0.05, Fig. 2E). The partial Mantel tests revealed no significant correlations between the internest averages of pairwise relatedness and the two behaviour indices AI and, separately MMAI, when controlling for geographic distance (partial Mantel test, AI, *r* = – 0.08, *P *= 0.58; MMAI, *r* = – 0.17, *P = *0.20) or between the geographic distance and AI and, separately MMAI, when controlling for internest averages of pairwise relatedness (partial Mantel test, AI, *r* = 0.17, *P *= 0.33; MMAI, *r* = 0.05, *P *= 0.81). The effect sizes of the sensitivity power analyses ranged between ≥ 0.30 and ≥ 0.47 (Supplementary Table 3). There was no significant correlation between the intranest averages of pairwise relatedness and any of the two aggression indices, AI and MMAI (Spearman rank correlation, AI, ρ = 0.24, *P *= 0.49; MMAI, ρ = 0.30, *P *= 0.37). Workers distinguished nestmates from non-nestmates, in that total antennation times were significantly shorter in intranest than non-aggressive internest encounters (two-sided *t*-test, *t*_183.62_ = – 5.69, *P* < 0.001).

## DISCUSSION

The genetic and behavioural data analysed here allow inferences on the social structure, the relationship between behaviour, relatedness, and geographic distance, the aggression level of workers, and the ability of non-aggressive workers to discriminate nestmates from non-nestmates. In all nests except one, the maximum number of alleles per locus was three, and one of these alleles was seen in all diploid workers of the same nest, likely originating from one single male, as expected under monogyny-monandry in a haplodiploid species. In the one nest mentioned, one new allele was detected, likely being a recent mutation (Schlick-Steiner et al. 2015). In the nests, H_O_ was significantly higher than H_E_, and we assume that unrelated gynes and males mated, leading to an increased heterozygosity and differing allele frequencies. The high global F_ST_ value (0.37) further indicates strong genetic differentiation among nests (Wright 1978). In combination with the high, significant pairwise F_ST_ values between nests (0.20–0.52; data not shown), limited or no gene flow can be inferred in our data, which in turn might increase genetic differentiation among nests. The F_ST_ value obtained here is comparable with those in various other studies applying a similar spatial sampling design ranging from several metres to kilometres, for example on *Anoplolepis gracilipes* (0.23, among colonies within a sampling region, Drescher et al. 2007), *Cataglyphis emmae* (0.27, 44 nests in a 1156 m^2^ plot, Jowers et al. 2013), and *Formica exsecta* (0.72, between pastures along a 6-km transect, Liautard & Keller 2001). It thus seems likely that mated *T. alpestre* gynes disperse and colonise distant new habitats as expected under monogyny (Heinze 2008), thus leading to increased genetic differentiation between nests.

The monogyny-monandry pattern was confirmed via the COLONY analysis, the high intranest average of pairwise relatedness (~ 0.75, Supplementary Table 4) in all nests, and the effective number of queens (*f)* of approximately one in all nests. The low internest averages of pairwise relatedness indicated that most workers were unrelated (Fig. 2E; Supplementary Table 4). The low *M*_e,p_ corroborated that queens had mated only once. The low NDE values indicated that all genotypes of all siring males were likely to have been detected, and the low NSE values that the overall sample size had probably been sufficient to detect all siring males. Based on these results, we assume that one nest represents one colony. Regarding Question (1), the results revealed monogynous-monandrous colonies in Tyrol, thus differing from the supercolony detected in Carinthia (Steiner et al. 2003). Furthermore, the results affirm a social polymorphism in *T. alpestre*, which is, besides *T. moravicum* with macrogynous-monogynous and microgynous-polygynous colonies (Schlick-Steiner et al. 2005), the second socially polymorphic species known from Palearctic *Tetramorium.*

While the observer of the behaviour encounters principally had access to the information of the origin of workers, we consider it unlikely that an observer bias had been introduced (van Wilgenburg & Elgar 2013). Among the authors of recent behaviour studies, there were both observers blind (Jongepier et al. 2015; Purcell et al. 2016; Yagound et al. 2016) and observers not blind about the origin of workers (Kleeberg & Foitzik 2016; Parmentier et al. 2016; Ślipiński & Żmihorski 2016). The aggression indices AI and MMAI yielded different values (Figs 2A–B and 3). AI integrates behaviour over time and is more balanced, in that brief aggressive interactions caused, for example, by disturbance (Huszar et al. 2014) do not strongly affect AI. MMAI uses the mean of the most aggressive behaviour observed and helps detecting if any aggression occurs. Thus, for MMAI to be high it suffices that workers attack each other at least briefly. The two indices were here used to detect both long-lasting and brief aggressive behaviours. The behavioural variation observed within nest pairings was likely due to individual responses of *T. alpestre* workers.

In the one-on-one internest encounters, we observed both aggressive and non-aggressive behaviour (Fig. 4). Regarding Question (2), we reveal that the nests sampled differ behaviourally. Moreover, the behavioural polymorphism is affirmed, as in only seven of 220 internest encounters, workers fought throughout the observation, while aggression was present but ceased in 62 encounters and was completely absent in 151 encounters, indicating that behaviour towards non-nestmates is a rather variable trait in *T. alpestre*. Aggressive behaviour was more likely to occur in internest than intranest encounters (Fig. 4), as workers generally attack non-nestmates to protect the nest against intruders (d’Ettorre & Lenoir 2009). Brief aggression observed in the first seconds of an encounter, however, might be a result of disturbance (Huszar et al. 2014) indicating that workers eventually recognised each other and stopped aggressive behaviour. Also the average of pairwise intranest relatedness had no influence on the average aggressive behaviour (Spearman rank correlation) implying that aggression, if occurring at all, seems not to depend on intranest relatedness, at least for this pilot study. As far as we know, peaceful behaviour between non-nestmates of monogynous colonies has been rarely observed in 24 species, for example in *Allomerus decemarticulatus, A. octoarticulatus* (Grangier et al. 2008), *Lasius austriacus* (Steiner et al. 2007 and references therein), *L. flavus* (Steinmeyer et al. 2012), and *Monomorium pharaonis* (Schmidt et al. 2010). In monogynous *T. alpestre* nests, peaceful behaviour might be explained by two hypotheses: (1) The colonies could stay monogynous on the long run, but workers might avoid aggression as it is time and energy consuming and linked to injury and mortality (Davies & Houston 1984; Cole 1986; Crozier 1987). The energy saved could be used for colony growth and reproduction. For example, the underground-living ant species *L. austriacus* tends mealybugs for honeydew; thus, foraging seems to be less important, and aggression can be reduced (Steiner et al. 2007). We assume that *T. alpestre* is similar in its trophic ecology and that, in principle, the same theory might apply. (2) The colonies might represent an intermediate state where aggression is being reduced, but polygyny and, eventually, supercoloniality have not yet been established (cf. Steiner et al. 2007). Regarding the unresolved transition from multicoloniality to supercoloniality, *T. alpestre* seems to be an ideal study species. Further research is needed to evaluate the two hypotheses and to unveil factors triggering transitions in social structure, behaviour, and genetic make-up of colonies.

We detected that neither the internest averages of pairwise relatedness nor the geographic distance correlated with worker behaviour (Mantel test). Only geographic distance and the internest averages of pairwise relatedness were significantly negatively correlated. Also the partial Mantel tests, controlling for an effect of another variable, revealed no significant correlation. Addressing Question (3), the results reveal that, at least in this pilot study in Tyrol, no significant correlation involving the behaviour was detectable, while with increasing geographic distance the internest averages of pairwise relatedness decreased. This decrease of pairwise relatedness is not unexpected as the distance among the most distant colonies was approximately 9 km. The sensitivity power analyses revealed that, with our sample size, alpha level, and power, correlation coefficients ranging between 0.30 and 0.47 would have yielded significant results (Supplementary Table 3), suggesting that moderate to strong correlations should have been detected despite the moderate sample size of this pilot study. At least for this study, the variation in behaviour seems not to be substantially spatially or genetically determined (unless the neutral variation detected by microsatellites is not representative of the variation encoding aggressive behaviour). Rather, the behavioural variation observed might depend on (i) the context as suggested, for example, for *L. austriacus* (Steiner et al. 2007), or (ii) the experience of workers in previous encounters with non-nestmates (Van Wilgenburg et al. 2010), or (iii) environmentally derived cuticular hydrocarbon cues (Liang & Silverman 2000). Furthermore, recent studies regarding correlations between behaviour and, for example, spatial distance revealed contrasting results: in some cases, spatial distance and aggressive behaviour were correlated (Pirk et al. 2001; Benedek & Kobori 2014; Frizzi et al. 2015; Fournier et al. 2016), while in others, no correlation was observed (Langen et al. 2000; van Wilgenburg 2007; Martin et al. 2012), corroborating the general need for further studies. A correlation known from other studies is one between aggressive behaviour and recognition cues (e.g. cuticular hydrocarbons, CHCs) allowing workers to discriminate nestmates from non-nestmates (Guerrieri et al. 2009; Fürst et al. 2012; Martin et al. 2012; Tsutsui 2013; di Mauro et al. 2015; Larsen et al. 2016). Recognition cues are genetically and/or environmentally determined (d’Ettorre & Lenoir 2009), but nestmate recognition seems not to be influenced by the social origin (monogyny vs polygyny) of workers (Rosset et al. 2007; Helanterä et al. 2011; Chirino et al. 2012). As there are only quantitative differences in CHCs of conspecific species, workers have to detect those small differences to correctly discriminate between nestmates and non-nestmates (di Mauro et al. 2015). Here, we did not analyse CHCs of *T. alpestre*, but we speculate that CHC profiles might be similar in Tyrolean nests, possibly leading to reduced aggressive behaviour as observed, for example, in *Formica exsecta* (Martin et al. 2012). However, analyses of CHCs are needed to evaluate this hypothesis for *T. alpestre*. Moreover, further studies are needed focusing on context-dependent behaviour and worker experience using specific behaviour assays, on chemical assays regarding chemical (dis)similarity using gas chromatography-mass spectrometry, and on comparative genomics and transcriptomics to identify and characterise potential variation in coding regions.

We detected that in non-aggressive encounters between non-nestmates, the total antennation time was significantly increased compared with intranest encounters, implying that workers distinguished nestmates from non-nestmates. This allows us to address Question (4): As mentioned above, a reduction in aggression probably increases time spent on tasks beneficial for the colony, such as nest maintenance and foraging. Lacking aggression is often associated with reduced recognition ability, for example in invasive species (Giraud et al. 2002). Although aggression was partly absent in *T. alpestre*, workers distinguished nestmates from non-nestmates, which was also observed in *Formica paralugubris* (Holzer et al. 2006) and in *L. austriacus* (Steiner et al. 2007), indicating that lacking aggression does not necessarily imply a loss of recognition ability.
