# Supplementary material for: An Alpine ant’s behavioural polymorphism: monogyny with and without internest aggression in Tetramorium alpestre
Source: Ethol Ecol Evol. 2017 Jul 20;30(3):220–34. doi: 10.1080/03949370.2017.1343868 (PMC5890305; doi:10.1080/03949370.2017.1343868)
Supplement: Supplementary Table 6 [file TEEE_A_1343868_SM7225.docx]

Supplementary Table 6.

MMAI values of the *T. alpestre* nests assayed.

|  | 17808 | 17809 | 17810 | 17811 | 17812 | 17813 | 17815 | 17816 | 17817 | 17818 | 17819 |
| --- | --- | --- | --- | --- | --- | --- | --- | --- | --- | --- | --- |
| 17808 | 0.00 |  |  |  |  |  |  |  |  |  |  |
| 17809 | 0.00 | 0.00 |  |  |  |  |  |  |  |  |  |
| 17810 | 3.00 | 1.50 | 0.00 |  |  |  |  |  |  |  |  |
| 17811 | 4.50 | 0.00 | 1.50 | 0.00 |  |  |  |  |  |  |  |
| 17812 | 0.00 | 0.00 | 2.50 | 0.00 | 0.00 |  |  |  |  |  |  |
| 17813 | 0.00 | 1.50 | 3.50 | 1.50 | 0.00 | 0.00 |  |  |  |  |  |
| 17815 | 1.75 | 3.25 | 0.00 | 3.75 | 2.75 | 4.25 | 0.00 |  |  |  |  |
| 17816 | 0.00 | 1.25 | 2.50 | 5.75 | 4.00 | 3.00 | 5.50 | 0.00 |  |  |  |
| 17817 | 0.00 | 0.00 | 0.00 | 0.00 | 0.00 | 0.00 | 2.25 | 0.00 | 0.00 |  |  |
| 17818 | 0.00 | 3.25 | 3.25 | 1.75 | 3.00 | 4.75 | 4.00 | 3.00 | 1.75 | 0.00 |  |
| 17819 | 1.25 | 0.00 | 1.25 | 0.00 | 0.00 | 5.25 | 3.75 | 3.00 | 1.50 | 0.00 | 1.50 |

The Mean maximum aggression index (MMAI, Vogel et al. 2009) is the arithmetic mean of the highest aggression values observed in each encounter over the four replicates.
