# Supplementary material for: An Alpine ant’s behavioural polymorphism: monogyny with and without internest aggression in Tetramorium alpestre
Source: Ethol Ecol Evol. 2017 Jul 20;30(3):220–34. doi: 10.1080/03949370.2017.1343868 (PMC5890305; doi:10.1080/03949370.2017.1343868)
Supplement: Supplementary Table 5 [file TEEE_A_1343868_SM7211.docx]

Supplementary Table 5.

AI values of the *T. alpestre* nests assayed.

|  | 17808 | 17809 | 17810 | 17811 | 17812 | 17813 | 17815 | 17816 | 17817 | 17818 | 17819 |
| --- | --- | --- | --- | --- | --- | --- | --- | --- | --- | --- | --- |
| 17808 | 0.00 |  |  |  |  |  |  |  |  |  |  |
| 17809 | 0.00 | 0.00 |  |  |  |  |  |  |  |  |  |
| 17810 | 0.04 | 0.01 | 0.02 |  |  |  |  |  |  |  |  |
| 17811 | 1.76 | 0.00 | 0.11 | 0.00 |  |  |  |  |  |  |  |
| 17812 | 0.00 | 0.00 | 0.02 | 0.00 | 0.00 |  |  |  |  |  |  |
| 17813 | 0.00 | 0.36 | 0.12 | 0.08 | 0.00 | 0.00 |  |  |  |  |  |
| 17815 | 1.22 | 0.01 | 0.00 | 0.03 | 0.03 | 1.06 | 0.00 |  |  |  |  |
| 17816 | 0.00 | 0.00 | 0.01 | 1.49 | 0.37 | 0.70 | 1.37 | 0.00 |  |  |  |
| 17817 | 0.00 | 0.00 | 0.00 | 0.00 | 0.00 | 0.00 | 0.01 | 0.00 | 0.00 |  |  |
| 17818 | 0.00 | 0.05 | 0.84 | 1.51 | 0.88 | 2.18 | 0.05 | 1.10 | 0.84 | 0.00 |  |
| 17819 | 0.05 | 0.00 | 0.00 | 0.00 | 0.00 | 0.06 | 0.13 | 0.03 | 0.00 | 0.00 | 0.11 |

For the Aggression index (AI, modified from d'Ettorre & Heinze 2005), the frequency of each observed aggression behaviour per worker was multiplied with its respective scoring level (4 to 7) and their sum divided by 180 as there were 180 records (one per second). The arithmetic mean of the four replicates was then calculated and used as AI.
