# Supplementary material for: An Alpine ant’s behavioural polymorphism: monogyny with and without internest aggression in Tetramorium alpestre
Source: Ethol Ecol Evol. 2017 Jul 20;30(3):220–34. doi: 10.1080/03949370.2017.1343868 (PMC5890305; doi:10.1080/03949370.2017.1343868)
Supplement: Supplementary Table 4 [file TEEE_A_1343868_SM7206.docx]

Supplementary Table 4.

Intranest and internest averages of pairwise relatedness values of the *T. alpestre* nests assayed.

|  | 17808 | 17809 | 17810 | 17811 | 17812 | 17813 | 17815 | 17816 | 17817 | 17818 | 17819 |
| --- | --- | --- | --- | --- | --- | --- | --- | --- | --- | --- | --- |
| 17808 | 0.65 |  |  |  |  |  |  |  |  |  |  |
| 17809 | – 0.02 | 0.78 |  |  |  |  |  |  |  |  |  |
| 17810 | – 0.05 | – 0.39 | 0.78 |  |  |  |  |  |  |  |  |
| 17811 | 0.24 | 0.01 | – 0.03 | 0.82 |  |  |  |  |  |  |  |
| 17812 | – 0.18 | – 0.13 | – 0.12 | – 0.07 | 0.70 |  |  |  |  |  |  |
| 17813 | 0.04 | 0.09 | – 0.12 | 0.31 | 0.16 | 0.76 |  |  |  |  |  |
| 17815 | – 0.18 | – 0.33 | 0.10 | – 0.12 | – 0.08 | – 0.06 | 0.73 |  |  |  |  |
| 17816 | 0.08 | 0.21 | – 0.18 | – 0.03 | – 0.23 | 0.01 | – 0.15 | 0.71 |  |  |  |
| 17817 | – 0.04 | – 0.02 | 0.17 | – 0.12 | – 0.17 | – 0.19 | 0.13 | – 0.05 | 0.62 |  |  |
| 17818 | – 0.26 | – 0.27 | – 0.15 | – 0.05 | 0.01 | – 0.07 | – 0.07 | – 0.33 | – 0.21 | 0.74 |  |
| 17819 | – 0.23 | 0.18 | – 0.14 | – 0.29 | – 0.01 | – 0.20 | – 0.33 | – 0.10 | – 0.03 | – 0.07 | 0.73 |

The intranest and internest averages of pairwise relatedness (*r*_ww_) were calculated in GenAlEx v6.502 using the algorithms of Queller and Goodnight (1989).
