# Supplementary material for: An Alpine ant’s behavioural polymorphism: monogyny with and without internest aggression in Tetramorium alpestre
Source: Ethol Ecol Evol. 2017 Jul 20;30(3):220–34. doi: 10.1080/03949370.2017.1343868 (PMC5890305; doi:10.1080/03949370.2017.1343868)
Supplement: Supplementary Table 3 [file TEEE_A_1343868_SM7196.docx]

Supplementary Table 3.

Calculated sensitivity power analyses.

| n | alpha | r | power Mantel test A | power Mantel test B | power Mantel test C | power Mantel test D | power Mantel test E |
| --- | --- | --- | --- | --- | --- | --- | --- |
| 55 | 0.05 | 0.10 | 0.12 | 0.13 | 0.18 | 0.18 | 0.29 |
| 55 | 0.05 | 0.11 | 0.12 | 0.14 | 0.20 | 0.20 | 0.31 |
| 55 | 0.05 | 0.12 | 0.13 | 0.15 | 0.23 | 0.22 | 0.34 |
| 55 | 0.05 | 0.13 | 0.14 | 0.16 | 0.25 | 0.24 | 0.37 |
| 55 | 0.05 | 0.14 | 0.15 | 0.17 | 0.27 | 0.27 | 0.40 |
| 55 | 0.05 | 0.15 | 0.16 | 0.18 | 0.29 | 0.30 | 0.43 |
| 55 | 0.05 | 0.16 | 0.17 | 0.20 | 0.32 | 0.32 | 0.46 |
| 55 | 0.05 | 0.17 | 0.19 | 0.22 | 0.34 | 0.35 | 0.49 |
| 55 | 0.05 | 0.18 | 0.21 | 0.24 | 0.37 | 0.38 | 0.52 |
| 55 | 0.05 | 0.19 | 0.22 | 0.26 | 0.40 | 0.41 | 0.55 |
| 55 | 0.05 | 0.20 | 0.23 | 0.28 | 0.43 | 0.44 | 0.57 |
| 55 | 0.05 | 0.21 | 0.25 | 0.30 | 0.46 | 0.47 | 0.60 |
| 55 | 0.05 | 0.22 | 0.27 | 0.32 | 0.49 | 0.50 | 0.63 |
| 55 | 0.05 | 0.23 | 0.28 | 0.35 | 0.52 | 0.53 | 0.65 |
| 55 | 0.05 | 0.24 | 0.30 | 0.37 | 0.55 | 0.55 | 0.68 |
| 55 | 0.05 | 0.25 | 0.33 | 0.39 | 0.57 | 0.58 | 0.70 |
| 55 | 0.05 | 0.26 | 0.36 | 0.41 | 0.60 | 0.61 | 0.73 |
| 55 | 0.05 | 0.27 | 0.38 | 0.44 | 0.63 | 0.64 | 0.75 |
| 55 | 0.05 | 0.28 | 0.41 | 0.46 | 0.66 | 0.67 | 0.77 |
| 55 | 0.05 | 0.29 | 0.43 | 0.49 | 0.69 | 0.70 | 0.78 |
| 55 | 0.05 | 0.30 | 0.46 | 0.51 | 0.71 | 0.72 | 0.80 |
| 55 | 0.05 | 0.31 | 0.49 | 0.53 | 0.74 | 0.75 | 0.82 |
| 55 | 0.05 | 0.32 | 0.51 | 0.55 | 0.76 | 0.77 | 0.83 |
| 55 | 0.05 | 0.33 | 0.54 | 0.57 | 0.78 | 0.79 | 0.85 |
| 55 | 0.05 | 0.34 | 0.57 | 0.59 | 0.81 | 0.81 | 0.86 |
| 55 | 0.05 | 0.35 | 0.60 | 0.60 | 0.83 | 0.83 | 0.87 |
| 55 | 0.05 | 0.36 | 0.62 | 0.62 | 0.84 | 0.85 | 0.88 |
| 55 | 0.05 | 0.37 | 0.64 | 0.64 | 0.86 | 0.87 | 0.89 |
| 55 | 0.05 | 0.38 | 0.66 | 0.65 | 0.88 | 0.88 | 0.90 |
| 55 | 0.05 | 0.39 | 0.69 | 0.67 | 0.89 | 0.90 | 0.91 |
| 55 | 0.05 | 0.40 | 0.71 | 0.69 | 0.90 | 0.91 | 0.92 |
| 55 | 0.05 | 0.41 | 0.73 | 0.70 | 0.92 | 0.92 | 0.93 |
| 55 | 0.05 | 0.42 | 0.75 | 0.72 | 0.93 | 0.93 | 0.94 |
| 55 | 0.05 | 0.43 | 0.77 | 0.74 | 0.94 | 0.94 | 0.95 |
| 55 | 0.05 | 0.44 | 0.78 | 0.75 | 0.95 | 0.95 | 0.96 |
| 55 | 0.05 | 0.45 | 0.79 | 0.77 | 0.96 | 0.96 | 0.97 |
| 55 | 0.05 | 0.46 | 0.80 | 0.78 | 0.96 | 0.96 | 0.97 |
| 55 | 0.05 | 0.47 | 0.81 | 0.80 | 0.97 | 0.97 | 0.98 |
| 55 | 0.05 | 0.48 | 0.82 | 0.81 | 0.98 | 0.98 | 0.99 |
| 55 | 0.05 | 0.49 | 0.82 | 0.83 | 0.98 | 0.98 | 0.99 |
| 55 | 0.05 | 0.50 | 0.83 | 0.85 | 0.98 | 0.98 | 0.99 |
| 55 | 0.05 | 0.51 | 0.84 | 0.86 | 0.99 | 0.99 | 0.99 |
| 55 | 0.05 | 0.52 | 0.85 | 0.87 | 0.99 | 0.99 | 1.00 |
| 55 | 0.05 | 0.53 | 0.86 | 0.88 | 0.99 | 0.99 | 1.00 |
| 55 | 0.05 | 0.54 | 0.87 | 0.89 | 0.99 | 0.99 | 1.00 |
| 55 | 0.05 | 0.55 | 0.88 | 0.90 | 0.99 | 0.99 | 1.00 |
| 55 | 0.05 | 0.56 | 0.90 | 0.91 | 0.99 | 0.99 | 1.00 |
| 55 | 0.05 | 0.57 | 0.91 | 0.92 | 1.00 | 1.00 | 1.00 |
| 55 | 0.05 | 0.58 | 0.92 | 0.92 | 1.00 | 1.00 | 1.00 |
| 55 | 0.05 | 0.59 | 0.94 | 0.93 | 1.00 | 1.00 | 1.00 |
| 55 | 0.05 | 0.60 | 0.95 | 0.94 | 1.00 | 1.00 | 1.00 |
| 55 | 0.05 | 0.61 | 0.96 | 0.95 | 1.00 | 1.00 | 1.00 |
| 55 | 0.05 | 0.62 | 0.97 | 0.95 | 1.00 | 1.00 | 1.00 |
| 55 | 0.05 | 0.63 | 0.98 | 0.96 | 1.00 | 1.00 | 1.00 |
| 55 | 0.05 | 0.64 | 0.99 | 0.97 | 1.00 | 1.00 | 1.00 |
| 55 | 0.05 | 0.65 | 0.99 | 0.98 | 1.00 | 1.00 | 1.00 |
| 55 | 0.05 | 0.66 | 0.99 | 0.98 | 1.00 | 1.00 | 1.00 |
| 55 | 0.05 | 0.67 | 1.00 | 0.99 | 1.00 | 1.00 | 1.00 |
| 55 | 0.05 | 0.68 | 1.00 | 0.99 | 1.00 | 1.00 | 1.00 |
| 55 | 0.05 | 0.69 | 1.00 | 0.99 | 1.00 | 1.00 | 1.00 |
| 55 | 0.05 | 0.70 | 1.00 | 1.00 | 1.00 | 1.00 | 1.00 |
| 55 | 0.05 | 0.71 | 1.00 | 1.00 | 1.00 | 1.00 | 1.00 |
| 55 | 0.05 | 0.72 | 1.00 | 1.00 | 1.00 | 1.00 | 1.00 |
| 55 | 0.05 | 0.73 | 1.00 | 1.00 | 1.00 | 1.00 | 1.00 |
| 55 | 0.05 | 0.74 | 1.00 | 1.00 | 1.00 | 1.00 | 1.00 |
| 55 | 0.05 | 0.75 | 1.00 | 1.00 | 1.00 | 1.00 | 1.00 |
| 55 | 0.05 | 0.76 | 1.00 | 1.00 | 1.00 | 1.00 | 1.00 |
| 55 | 0.05 | 0.77 | 1.00 | 1.00 | 1.00 | 1.00 | 1.00 |
| 55 | 0.05 | 0.78 | 1.00 | 1.00 | 1.00 | 1.00 | 1.00 |
| 55 | 0.05 | 0.79 | 1.00 | 1.00 | 1.00 | 1.00 | 1.00 |
| 55 | 0.05 | 0.8 | 1.00 | 1.00 | 1.00 | 1.00 | 1.00 |
| 55 | 0.05 | 0.81 | 1.00 | 1.00 | 1.00 | 1.00 | 1.00 |
| 55 | 0.05 | 0.82 | 1.00 | 1.00 | 1.00 | 1.00 | 1.00 |
| 55 | 0.05 | 0.83 | 1.00 | 1.00 | 1.00 | 1.00 | 1.00 |
| 55 | 0.05 | 0.84 | 1.00 | 1.00 | 1.00 | 1.00 | 1.00 |
| 55 | 0.05 | 0.85 | 1.00 | 1.00 | 1.00 | 1.00 | 1.00 |
| 55 | 0.05 | 0.86 | 1.00 | 1.00 | 1.00 | 1.00 | 1.00 |
| 55 | 0.05 | 0.87 | 1.00 | 1.00 | 1.00 | 1.00 | 1.00 |
| 55 | 0.05 | 0.88 | 1.00 | 1.00 | 1.00 | 1.00 | 1.00 |
| 55 | 0.05 | 0.89 | 1.00 | 1.00 | 1.00 | 1.00 | 1.00 |
| 55 | 0.05 | 0.90 | 1.00 | 1.00 | 1.00 | 1.00 | 1.00 |
| 55 | 0.05 | 0.91 | 1.00 | 1.00 | 1.00 | 1.00 | 1.00 |
| 55 | 0.05 | 0.92 | 1.00 | 1.00 | 1.00 | 1.00 | 1.00 |
| 55 | 0.05 | 0.93 | 1.00 | 1.00 | 1.00 | 1.00 | 1.00 |
| 55 | 0.05 | 0.94 | 1.00 | 1.00 | 1.00 | 1.00 | 1.00 |
| 55 | 0.05 | 0.95 | 1.00 | 1.00 | 1.00 | 1.00 | 1.00 |
| 55 | 0.05 | 0.96 | 1.00 | 1.00 | 1.00 | 1.00 | 1.00 |
| 55 | 0.05 | 0.97 | 1.00 | 1.00 | 1.00 | 1.00 | 1.00 |
| 55 | 0.05 | 0.98 | 1.00 | 1.00 | 1.00 | 1.00 | 1.00 |
| 55 | 0.05 | 0.99 | 1.00 | 1.00 | 1.00 | 1.00 | 1.00 |
| 55 | 0.05 | 1.00 | 1.00 | 1.00 | 1.00 | 1.00 | 1.00 |

Analyses were calculated using the “mantelPower”-function in the “biotools”-package in R. n represents the total sample size, alpha the applied alpha, r the incrementing effect size, and power the computed power for the mantel tests: power Mantel test A represents the values for the power Mantel test geographic distance and AI, power Mantel test B for geographic distance and MMAI, power Mantel test C for the averages of pairwise intranest relatedness and AI, power Mantel test D for the averages of pairwise intranest relatedness and MMAI, and power Mantel test E for the averages of pairwise intranest relatedness and geographic distance. Values highlighted represent the power 0.8 and its respective effect size for each simulated power Mantel test.
