# Supplementary material for: An Alpine ant’s behavioural polymorphism: monogyny with and without internest aggression in Tetramorium alpestre
Source: Ethol Ecol Evol. 2017 Jul 20;30(3):220–34. doi: 10.1080/03949370.2017.1343868 (PMC5890305; doi:10.1080/03949370.2017.1343868)
Supplement: Supplementary Table 2 [file TEEE_A_1343868_SM7182.docx]

Supplementary Table 2.

Geographic distances between the *T. alpestre* nests assayed.

|  | 17808 | 17809 | 17810 | 17811 | 17812 | 17813 | 17815 | 17816 | 17817 | 17818 | 17819 |
| --- | --- | --- | --- | --- | --- | --- | --- | --- | --- | --- | --- |
| 17808 | 0.00 |  |  |  |  |  |  |  |  |  |  |
| 17809 | 2.22 | 0.00 |  |  |  |  |  |  |  |  |  |
| 17810 | 0.01 | 2.22 | 0.00 |  |  |  |  |  |  |  |  |
| 17811 | 0.09 | 2.28 | 0.10 | 0.00 |  |  |  |  |  |  |  |
| 17812 | 0.08 | 2.27 | 0.09 | 0.01 | 0.00 |  |  |  |  |  |  |
| 17813 | 1.33 | 1.22 | 1.33 | 1.42 | 1.41 | 0.00 |  |  |  |  |  |
| 17815 | 2.29 | 0.08 | 2.29 | 2.36 | 2.35 | 1.27 | 0.00 |  |  |  |  |
| 17816 | 2.28 | 0.07 | 2.28 | 2.35 | 2.34 | 1.27 | 0.01 | 0.00 |  |  |  |
| 17817 | 2.24 | 0.02 | 2.23 | 2.30 | 2.29 | 1.24 | 0.07 | 0.06 | 0.00 |  |  |
| 17818 | 8.91 | 6.87 | 8.90 | 8.98 | 8.97 | 7.59 | 6.79 | 6.80 | 6.86 | 0.00 |  |
| 17819 | 8.89 | 6.85 | 8.88 | 8.97 | 8.96 | 7.57 | 6.77 | 6.78 | 6.83 | 0.05 | 0.00 |

GenAlEx v6.502 (Peakall & Smouse 2012) was used to calculate pairwise geographic distances between nests. All values are given in kilometres.
