# Supplementary material for: An Alpine ant’s behavioural polymorphism: monogyny with and without internest aggression in Tetramorium alpestre
Source: Ethol Ecol Evol. 2017 Jul 20;30(3):220–34. doi: 10.1080/03949370.2017.1343868 (PMC5890305; doi:10.1080/03949370.2017.1343868)
Supplement: Supplementary Table 1 [file TEEE_A_1343868_SM7172.docx]

Supplementary Table 1.

Characteristics of the *T. alpestre* nests assayed.

| Nest | Latitude | Longitude | Elevation  [m a.s.l.] | *Q*_est_ | *M*_est_ | *f* |
| --- | --- | --- | --- | --- | --- | --- |
| 17808 | 47.2097 N | 11.1025 E | 1950 | 1 | 1 | 1.21 |
| 17809 | 47.2192 N | 11.0769 E | 1817 | 1 | 1 | 0.95 |
| 17810 | 47.2096 N | 11.1024 E | 1948 | 1 | 1 | 0.95 |
| 17811 | 47.2099 N | 11.1036 E | 1938 | 1 | 1 | 0.89 |
| 17812 | 47.2099 N | 11.1035 E | 1940 | 1 | 1 | 1.10 |
| 17813 | 47.2097 N | 11.0847 E | 1686 | 1 | 1 | 0.98 |
| 17815 | 47.2192 N | 11.0756 E | 1803 | 1 | 1 | 1.04 |
| 17816 | 47.2192 N | 11.0758 E | 1806 | 1 | 1 | 1.07 |
| 17817 | 47.2194 N | 11.0767 E | 1819 | 1 | 1 | 1.28 |
| 17818 | 47.2208 N | 10.9858 E | 1759 | 1 | 1 | 1.03 |
| 17819 | 47.2214 N | 10.9861 E | 1769 | 1 | 1 | 1.04 |

*Q_est_* and *M_est_* are the estimated numbers of queens and males, respectively, based on the results of COLONY (Jones & Wang 2010). The effective number of queens (*f*) was calculated following Pamilo (1991).
