# Supplementary material for: An Alpine ant’s behavioural polymorphism: monogyny with and without internest aggression in Tetramorium alpestre
Source: Ethol Ecol Evol. 2017 Jul 20;30(3):220–34. doi: 10.1080/03949370.2017.1343868 (PMC5890305; doi:10.1080/03949370.2017.1343868)
Supplement: Supplementary Material [file TEEE_A_1343868_SM0043.docx]

Supplementary Material

*Field work and worker maintenance*

Workers were killed in 96% ethanol and stored at – 20 °C for genetic analyses. Living workers were collected without skin contact using an aspirator and kept in polypropylene boxes (12 × 12 cm and 6 cm height) at room temperature (approx. 25 °C). A drop of honey water on a 10-Euro-cent coin, tap water in a test tube plugged with tissue paper as a drinking aid, and paper tissue as a hiding place were presented to the workers. Every other day, the honey water, water, and paper tissue were changed to prevent mould formation. The walls of the boxes were Fluon-coated (GP1, De Monchy International BV, Rotterdam, Netherlands) to prevent escape.

*Microsatellite genotyping and allele analyses*

The nine primer pairs used (51b, 51d, 51i, 51o, 52a, 55a, 56d, 56h, and 57l) had been developed for *Tetramorium* sp. E and *T. tsushimae* (Steiner et al. 2008). PCR for genotyping was done in 5 µL reaction volume with 0.5 µL template DNA, 1 × reaction buffer (Bioline, UK), 0.2 µM fluorescent-labelled M13 primer, 0.02 µM M13 tailed locus specific forward primer, 0.2 µM untailed specific reverse primer, and 0.125 U MyTaq polymerase (Bioline) on a UnoCycler 1200 (VWR, Radnor, USA). Cycling conditions were 94 °C for 5 min followed by 35 cycles at 94 °C for 30 sec, 60 °C for 1 min, 72 °C for 45 sec, and a final extension at 68 °C for 20 min.

Fragment analysis was carried out on an ABI3730XL genetic analyser (Applied Biosystems, Foster City, USA) by a commercial provider (Comprehensive Cancer Center DNA Sequencing & Genotyping Facility, University of Chicago, USA). Traces were visualized using PeakScanner software v1.0 (Applied Biosystems) and scored manually. The allelic dropout rates and error rates in COLONY (Jones & Wang 2010) were each set to 0.05, and three runs using different random number seeds were performed, checking the reliability of the results as recommended by the user’s manual.

*One-on-one encounters*

One-on-one worker encounters were performed following Giraud et al. (2002) using clean glass vials of 15.75 mm inner diameter with Fluon-coated walls. Films were recorded for 3 min each using a high-definition camera (Handycam HDR-XR 155, Sony, Tokyo, Japan) and the labels identifying worker origin were filmed throughout the whole encounter to reduce possibility of confusion. All video recordings of the one-on-one encounters can be obtained upon request.
